# Development of resveratrol-loaded hollow mesoporous silica enhances mechanical performance and biocompatibility of dental resin cement

**DOI:** 10.1186/s11671-026-04532-7

**Published:** 2026-04-10

**Authors:** Huiyi Yan, Chan He, Wenan Peng, Chuliang Tang, Chenmin Yao, Hongye Yang, Cui Huang

**Affiliations:** 1 State Key Laboratory of Oral & Maxillofacial Reconstruction and Regeneration, Key Laboratory of Oral Biomedicine Ministry of Education, Hubei Key Laboratory of Stomatology, School & Hospital of Stomatology, Wuhan University, Wuhan, China; 2 Fujian Key Laboratory of Oral Diseases, Fujian Provincial Engineering Research Center of Oral Biomaterial, Stomatological Key lab of Fujian College and University, School and Hospital of Stomatology, Fujian Medical University, Fuzhou, China

**Keywords:** Resveratrol, Mesoporous silica, Dental, Resin cement, Cytotoxicity

## Abstract

**Objectives:**

This work aims to develop a novel filler system by using resveratrol-loaded hollow mesoporous silica (HMS) to enhance the mechanical properties and reduce cytotoxicity of dental resin cement.

**Materials & Methods:**

HMS was synthesised via an ammonia-catalysed sol–gel process. Resveratrol (Res) was encapsulated by HMS to obtain Res@HMS. Res@HMS was characterised via transmission electron microscopy, dynamic light scattering, nitrogen adsorption/desorption measurements, fourier transform infrared spectroscopy and thermogravimetric analysis. An experimental resin cement containing Res@HMS was prepared. The mechanical properties of resin cement were measured, including flexural strength, elastic modulus and Shore hardness. The in vitro cytotoxicity of resin cement was evaluated via CCK8, lactate dehydrogenase assay and reactive oxygen species measurement.

**Results:**

A Res@HMS filler system with hollow mesoporous structure and the ability to encapsulate large amounts of resveratrol was successfully synthesised. This experimental resin cement modified by Res@HMS exhibited remarkable mechanical properties, especially against thermocycling ageing. The introduction of Res@HMS effectively protected human gingival fibroblasts against resin monomer-induced cytotoxicity.

**Conclusion:**

Res@HMS can serve as a versatile modifier to optimise dental resin cement. This modification can preserve the long-term mechanical properties of dental cement and also reduce resin monomer-induced cytotoxicity.

**Clinical significance:**

The effective integration of mesoporous nanoparticles with plant extracts offers a novel filler that promises to balance the mechanical properties and cytotoxicity of resin cement, paving the way for developing a new generation of dental restorative materials.

## Introduction

Ceramics are widely used in dental restoration because of their desirable mechanical properties and superior aesthetics [1]. Resin cement, which allows ceramics bond to tooth structure, has become the key for clinical success of dental restoration. The short-term bond strength of cement–dentin interfaces is satisfactory. However, the long-term stability and biocompatibility of resin cements remain key limitations, which can affect the clinical longevity of indirect restorations [2].

Indeed, organic/inorganic hybrid materials have emerged as a classic design concept for health applications, exemplified by MXene, CoFe_2_O_4_, and bacterial nanocellulose composites for health monitoring [3], halloysite/wax Pickering emulsion coatings for suture modification [4], and nitrogen-doped carbon dots-copper oxide nanoparticle composites for antibacterial treatment [5]. Similarly, in the field of dentistry, by dispersing inorganic filler particles in a resin matrix, resin cement can be achieved that exhibit both excellent mechanical properties and multiple functionalities. The interaction between resin matrix and inorganic particles must be strong enough to ensure long-term cementation. A conventional method adopted to establish this interaction is by modifying the filler surface with silane treatment. However, silanol coupling always hydrolyses over time. As a result, the formation of micromechanical interlocking via the infiltration of resin matrix into porous fillers has drawn wide interest. Porous fillers can endow the resin composites with wear resistance [6], improved mechanical properties [7] and enhanced finishing ability [8]. Aside from mechanical effects, porous structures can also exert bioactivity by releasing biological constituents from the pores. Mesoporous nanoparticles have attracted increased attention because of its high specific surface area, tailorable and stable framework and satisfactory biocompatibility [9]. Among them, hollow mesoporous silica (HMS) has been proved a desirable carrier of active substances, such as drugs in polymeric biomaterials [10, 11].

Another crucial aspect of resin cement lies in their biocompatibility, as the potential release of substances can have adverse effects on oral health, including post-cementation hypersensitivity, gingivitis, or even pulpal necrosis [12]. Previous studies have demonstrated the in vitro cytotoxicity of resin cement. Unpolymerised monomers, such as bisphenol A glycerolate dimethacrylate (BisGMA), triethylene glycol dimethacrylate (TEGDMA), 2-hydroxyethyl methacrylate (HEMA) and urethane dimethacrylate (UDMA), would leach from resin cement and cause allergic or toxic effects [13, 14]. These effects could induce cellular inflammatory response or toxic oxidative stress, leading to hypersensitivity or postoperative pain. Thus, appropriate ingredients should be introduced into the resin cement to improve its biocompatibility.

**Fig. 1:**
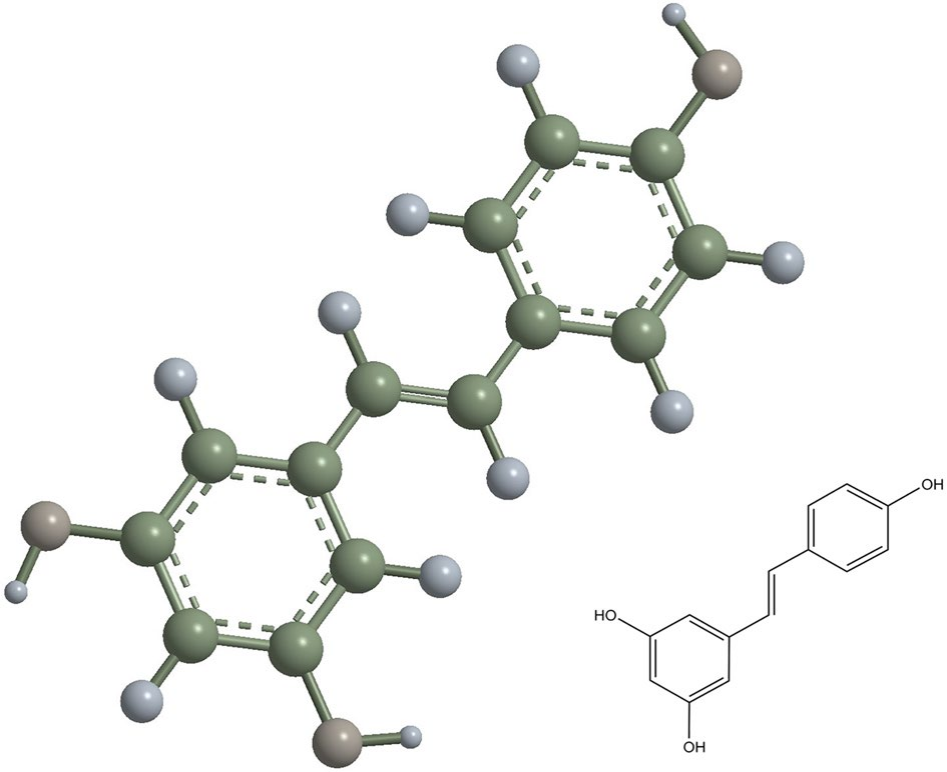
Chemical structure illustration of resveratrol (trans-3,5,4′-trihydroxystilbene)

Polyphenol antioxidants have been used to protect oral fibroblasts from oxidative stress and cytotoxicity [15]. Resveratrol, a natural dietary polyphenolic phytoalexin, is abundantly found in grapes, berries, nuts and other plant extracts. It exists in two isomeric forms, of which the trans- form (trans-3,5,4′-trihydroxystilbene) possesses significant biologically activity (Fig. 1). Resveratrol is widely used in biomedical fields because of its anticancer, anti-inflammatory, antioxidative activity and cardioprotective effects [16, 17]. In previous studies, resveratrol extract was directly used to modify bonding agent and reduce the production of reactive oxygen species and DNA damage [18]. However, this method remains inapplicable for dental clinical practice. Moreover, sustained release of resveratrol is difficult to achieve under this circumstance, thereby minimising its protective effects against cytotoxicity.

In this study, we hypothesised that HMS may act as both porous fillers and promising carriers for resveratrol in resin cement. The mechanical performance of resin cement may be improved through physical interlocking between porous structure and resin matrix, while the cytotoxicity may be reduced by sustained release of resveratrol, respectively. To the best of our knowledge, no study on the application of resveratrol-loaded HMS in resin cement modification has been reported until now. Accordingly, a resveratrol-encapsulated hollow mesoporous silica (Res@HMS) was synthesised and characterised in this study. An experimental resin cement containing Res@HMS was prepared. The null hypotheses are (1) Res@HMS cannot enhance the mechanical properties of resin cement (2) Res@HMS cannot confer protect effects against resin cement-induced cytotoxicity.

## Materials and methods

### Materials

Tetraethyl orthosilicate (TEOS, 98%), ammonium hydroxide (28–30 wt%) and ethanol (99%) were purchased from Sinopharm Chemical Reagent Co., Ltd. (Shanghai, China). BisGMA, TEGDMA (95%), camphorquinone (97%), resveratrol (trans-3,5,4′-trihydroxystilbene, 98%) and 2′,7′-dichlorofluorescin diacetate (DCF-DA) were obtained from Sigma-Aldrich (Milwaukee, USA). Amine-modified and positively charged polystyrene microspheres was procured from DAE Scientific Co. Ltd. (Tianjin, China). Foetal bovine serum (FBS) and α-modified minimal essential medium (α-MEM) were acquired from Hyclone, Logan (UT, USA). Then, 1% penicillin/streptomycin was bought from Amresco LLC, Solon (OH, USA). Cell Counting Kit-8 (CCK8) was obtained from Dojindo (Tokyo, Japan). Lactate dehydrogenase (LDH) assay kit (A020-2-2) was purchased from Nanjing Jiancheng Bioengineering Institute (Nanjing, China). Human reactive oxygen species ELISA kit was obtained from Applygen Technologies Co. Ltd. (Beijing, China). Deionised water was prepared by our own laboratory. All these chemicals were used without further purification.

### Methods

#### Synthesis of Res@HMS

**Fig. 2:**
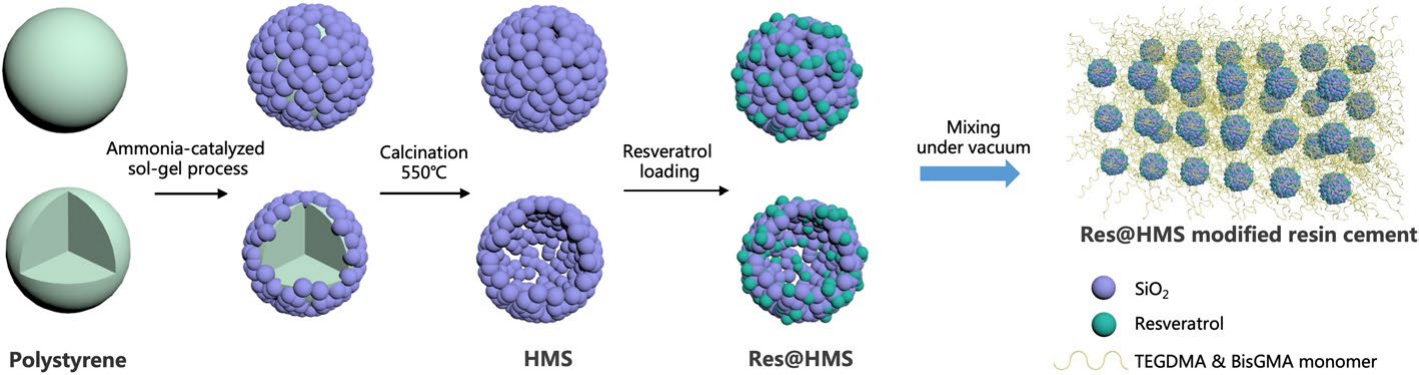
Schematic summarizing the development of Res@HMS and the optimization of dental resin cement by Res@HMS

The schematic for synthesising Res@HMS is demonstrated in Fig. 2. Firstly, HMS was synthesised via an ammonia-catalysed sol–gel process [19]. In particular, 250 mg of amine-modified positively charged polystyrene microspheres was added into 30 mL ethanol under ultrasonic dispersion. The polystyrene suspension was heated to 50 °C under stirring at the rate of 100 rpm, followed by the addition of 2.25 mL ammonium hydroxide solution. Afterwards, 0.75 g TEOS was rapidly incorporated into the solution and stirred for 1 h. The suspension was centrifuged at 4000 rpm and then washed with deionised water and ethanol thrice. HMS was obtained in a subsequent step by calcinating the polystyrene template at 550 °C for 8 h.

The loading of resveratrol into HMS was performed as follows. Resveratrol/ethanol solution (10 mg/mL) was prepared in advance, and then mixed with 150 mg HMS, sonicated for 15 min and gently shaken for 24 h. The mixture (Res@HMS) was centrifuged, washed with deionised water, vacuum-dried and stored at 4 °C until use.

#### Preparation of dental resin cement

The original resin cement was prepared according to a common formulation with photoinitiators. Specifically, BisGMA and TEGDMA were mixed at a mass ratio of 1:1. Subsequently, 1% (all mass fractions) of the photoinitiator of camphorquinone was added, thus forming a light-curable resin matrix. Resin cement without fillers served as the control.

The experimental resin cement was reinforced with fillers (Res@HMS). Firstly, Res@HMS was premixed with resin matrix at the mass fraction of 10% on a shaking table. The mixture was agitated overnight on a magnetic stirrer at room temperature in the dark until a flowable paste formed. All pastes were placed in a vacuum oven for 12 h to remove air bubbles, and to allow the complete infiltration of resin monomers into mesopores and hollow spaces of Res@HMS. Subsequently, each resin cement was placed in an appropriate mould covered with Mylar strip and polymerised with a curing unit (Triad 2000, Dentsply, York, PA) for 60 s on each side. All cured specimens were demoulded, burnished with carborundum papers and stored in distilled water at 37 °C for 24 h.

### Characterisation

#### Characterisation of porous fillers

1. Morphology and size of Res@HMS

The morphology of nanoparticles was characterized using a transmission electron microscopy (TEM, HT7700, Hitachi, Japan). The analysis was conducted using an accelerating voltage of 120 kV under high vacuum mode. For sample preparation, the powder was ultrasonically dispersed in absolute ethanol for 10 min. A droplet of the suspension was then deposited onto a carbon-film-coated copper grid and dried at room temperature prior to observation. Dynamic Light Scattering (DLS) analysis was carried out using a Malvern instrument at 25 °C. The measurement employed a He-Ne laser with a wavelength of 633 nm and a backscatter detection angle of 173°.

(2) Specific surface area of Res@HMS

Nitrogen adsorption/desorption analysis was typically performed using an ASAP 2020 analyser (Micromeritics Instrument Corp., USA). The measurement was conducted at 77 K using a liquid nitrogen bath. Prior to analysis, the sample was degassed under vacuum at 120 °C for at least 6 h to remove adsorbed substances. The specific surface area was calculated using the Brunauer–Emmett–Teller (BET) method within the relative pressure (P/P₀) range of 0.05–0.30, and the pore size distribution was derived from the adsorption branch of the isotherm using the Barrett–Joyner–Halenda (BJH) method.

(3) Evaluation of resveratrol loading into HMS

The functional groups in the Res@HMS were characterised via fourier transform infrared (FTIR) spectroscopy by using a Nicolet 5700 spectrometer (Thermo Scientific Inc., Madison, WI, USA). The spectra were recorded from 400 to 4000 cm^− 1^ at a resolution of 4 cm^− 1^. The average weight% of resveratrol on HMS was analysed using a thermogravimetric analyser (Perkin Elmer TGA-6, Thermo Electron Corp. USA). The measurement was performed under a nitrogen atmosphere with a constant gas flow rate of 20 mL/min. The sample (approximately 5–10 mg) was placed in an open alumina crucible and heated from room temperature to 600 °C at a rate of 10 °C/min.

#### Mechanical properties of resin cement

1. Flexural strength

The resin cement was placed in a mould with a dimension of 2 mm × 2 mm × 25 mm and covered with a Mylar strip. Each specimen was photocured, demoulded, polished with carborundum papers and stored in a 37 °C water bath for 24 h; 10 specimens randomly selected from each group were applied to demonstrate 24 h mechanical properties. The remaining 10 specimens from each group were placed in a thermal cycling machine from 5 to 55 °C for 10,000 cycles at a dwell time of 15 s to test the ageing performance of the specimens. For three-point bending testing, a span of 10 mm and a crosshead speed of 1 mm/min were used to fracture the specimens by using a computer-controlled universal testing machine (E1000, Instron, US). Accordingly, Flexural strength and Elastic modulus was calculated.

(2) Shore hardness of resin cement

Five specimens of each resin cement were prepared for the Shore hardness test. Each flowable paste was poured into a mould with a dimension of 6 mm × 40 mm × 40 mm and photocured for 1 min at every 2 mm thickness until the specimens were manufactured to the height of 6 mm. The specimens were removed from the mould and stored in a 37 °C water bath for 24 h before measurements. Regular intervals were set at least 12 mm from the edge of the specimens and 10 mm apart [20]. Dwell time was set to 5 s, and Shore hardness tests were performed using a Shore hardness tester (LX-D, Sundoo, China). The measurements were repeated at five points for each specimen (*n* = 25 each group). Afterwards, the specimens were placed in a thermal cycling machine from 5 to 55 °C for 10,000 cycles with a dwell time of 15 s to form thermal cycling-aged specimens. The Shore hardness of each specimen was tested again.

#### *In vitro* bioactivity of resin cement

1. Preparation of resin cement extracts

A polytetrafluoroethylene mould with circular holes (Ø 5 mm, H 2 mm) was used as a model for specimen preparation. The extracts were prepared as follows. First, 10 µL resin cements with or without Res@HMS were dripped into the moulds and light cured for 1 min. Two specimens from each group were removed from the mould and stored in a 37 °C water bath for 24 h. The specimens were then submerged in 10 mL supplemented α-MEM and incubated under the same cell conditions. Afterwards, 3 mL cement extract medium of each specimen was collected sequentially at time points of 1, 5 and 10 d, then filtered through a 0.22 μm syringe filter. Then the extracts were used to evaluate the protective effects of Res@HMS on cells over time.

(2) CCK8 and LDH assays

Human gingival fibroblasts (HGFs) HGFs were purchased from Zhong Qiao Xin Zhou Biotechnology Co., Ltd (China). HGFs were seeded in 96-well plates (5000 cells per well) for 24 h to ensure the adherence of the cells on the well surface. The culture medium is α-MEM containing 10% FBS and 1% penicillin/streptomycin at 37 °C and 5% CO_2_. Subsequently, 10 µL of cement extracts from different groups was exposed to the cells after 1 h (the extracts were diluted with a medium at the ratio of 1:10). After incubation for 24 h, the culture supernatants were harvested for LDH assay, and the seeded cells were cultured with 110 µL of CCK8 solution in darkness for 4 h at 37 °C. Absorbance was measured at 450 nm by using a microplate reader. Percent survivals were plotted as the ratio to the values of control group to test the protective effects of Res@HMS against cement-induced cytotoxicity. CCK8 assay was implemented in sextuplicate. Cell viability was assessed through the extracellular leakage of LDH by using an LDH assay kit. Absorbance was measured at 450 nm, and LDH assay was implemented in sextuplicate.

(3) Reactive oxygen species (ROS) measurement

Intracellular generation of ROS was monitored using a human ROS ELISA kit. HGFs were seeded on slides (10,000 cells per slide) for 24 h before cultured in different cement extract media (diluted with medium at the ratio of 1:10) for another 24 h. Afterwards, the media were pipetted out, and the cells were washed with phosphate buffer solution (PBS) three times. The HGFs were then incubated with 10 µM DCFH-DA under darkness at 37 °C for 30 min. Prior to observations, the samples were gently washed with PBS three times and placed into a cassette. ROS was determined using a fluorescence microscope (DP71, OLYMPUS, China) with a blue filter at the excitation wavelength of 488 (500 ± 15) nm. Five fields for each sample were observed under the same conditions.

#### Statistical analysis

SPSS (IBM SPSS Statistics 22.0, Armonk, NY, USA) was used for statistical analysis. Statistical differences in flexural strength, elastic modulus and Shore hardness between the control and experimental groups were interpreted by two-way ANOVA and post-hoc Tukey’s test. One-way ANOVA and post-hoc Tukey’s test were applied to analyse CCK8 assay and LDH activity results. Statistical significance was defined as *p* < 0.05.

## Results

### Characterisation of HMS and Res@HMS

**Fig. 3:**
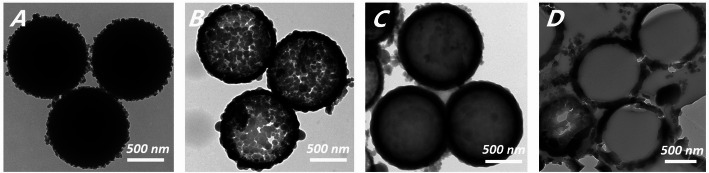
Representative TEM images of silica-coated polystyrene micro spheres (**A**), HMS (**B**), Res@HMS (**C**), and ultrathin section of epoxy resin-embedded Res@HMS (**D**)

**Fig. 4:**
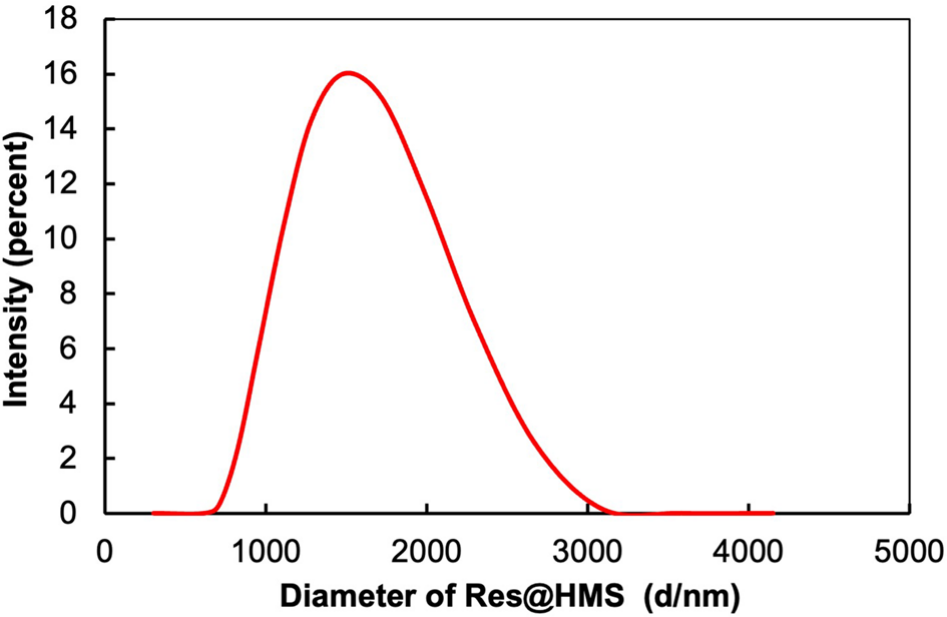
Diameter distribution of Res@HMS calculated by dynamic light scattering (DLS)

HMS was successfully prepared via a sol-gel method by using polystyrene microspheres as template. The TEM images in Fig. 3 show that small protuberances protruded on the surface of polystyrene microspheres after ammonia-catalysed process, indicating that the microspheres were coated with silica layers (Fig. 3A). As shown in Fig. 3B, HMS was well-dispersed with the diameter of 1 μm, and the calcination of the polystyrene template resulted in microspheres acquiring a hollow structure, with distinct pores on the shell. Nitrogen adsorption/desorption measurements revealed that the average surface area of HMS was 36.73 m^2^/g, whereas the average pore volume was 0.08565 cm^3^/g with a pore size of 9.328 nm. After resveratrol was encapsulated into the HMS, the porous space became blurry, as shown in Fig. 3C. An ultramicrotome was used to prepare an ultrathin section of epoxy resin-embedded Res@HMS to examine in greater detail the morphology of Res@HMS. TEM images (Fig. 3D) show large cavities inside Res@HMS, and the thickness of the shell was about 100 nm. DLS demonstrated the diameter distribution of Res@HMS (Fig. 4), with an average diameter of 1186 nm.

### **Evaluation of resveratrol loading into HMS**

**Fig. 5:**
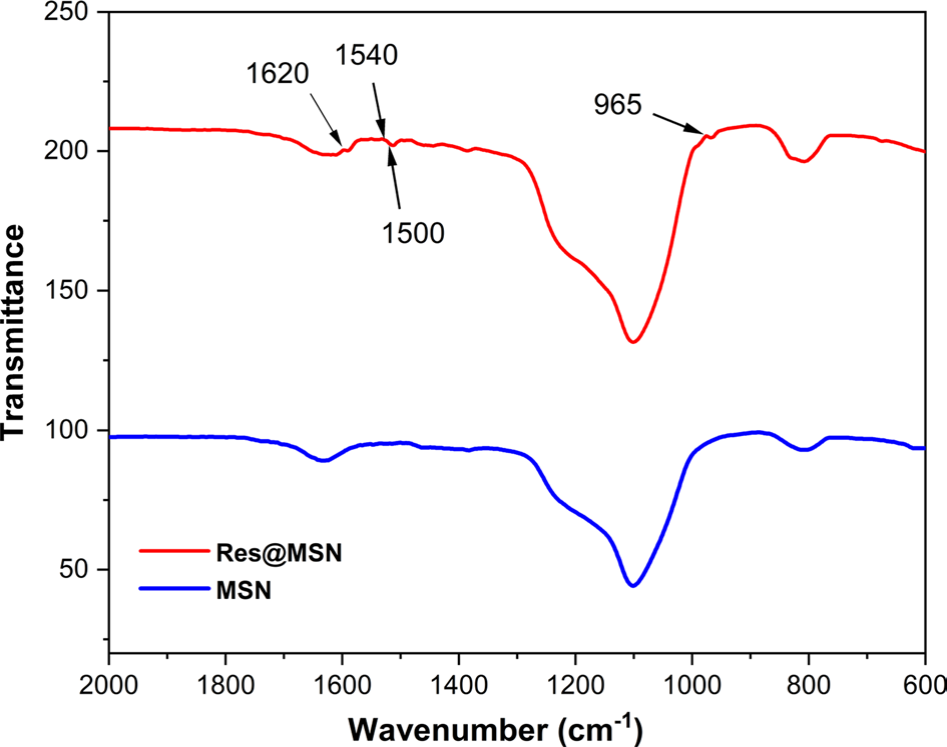
FTIR spectra of HMS and Res@HMS

The spectra of Res@HMS were analysed via FTIR spectroscopy and TGA to assess whether resveratrol was successfully loaded in HMS. A comparison of the FTIR spectra of HMS and Res@HMS revealed that some changes in HMS occurred after resveratrol encapsulation (Fig. 5). For pure HMS, typical absorbance peaks were observed, such as Si–O–Si bending vibration at 804 cm^− 1^, Si–OH bending vibration at 960 cm^− 1^, Si–O stretching vibration at 1070 cm^− 1^ and bending vibration peak of hydroxyl group at 1638 cm^− 1^. For Res@HMS, benzene skeleton stretching peaks at 1620, 1540 and 1500 cm^− 1^, as well as specific absorbing peak of –C = C– at 965 cm^− 1^ from resveratrol, were observed. The absence of obvious band shifts in the resveratrol vibrations after loading indicates that resveratrol is primarily loaded into HMS via physical adsorption, such as pore confinement, van der Waals forces, and possible hydrogen bonding.

**Fig. 6:**
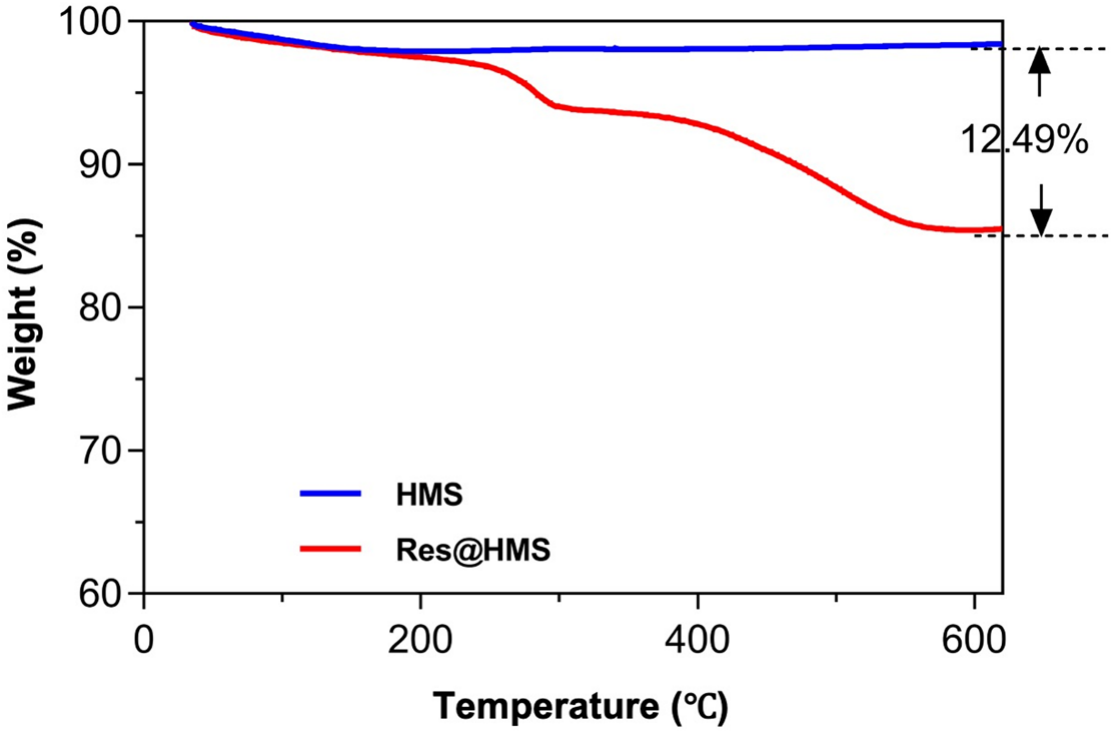
TGA spectra of HMS and Res@HMS

The TGA curves (Fig. 6) show that both HMS and Res@HMS exhibit similar mass loss in the temperature range of 25–150 °C, which is attributed to the evaporation of physically adsorbed water molecules on the nanoparticles [21]. It is worth noting that Res@HMS shows a distinct mass loss in the range of 400–600 °C, which represents the mass fraction of Res loaded in the HMS, approximately 12.49%.

### Mechanical properties of resin cement

The mechanical properties of resin cement with or without Res@HMS are shown in Table 1. Results revealed that the addition of Res@HMS significantly increased the elastic modulus of resin cement from 1.59 GPa up to 2.24 GPa (*p* < 0.05). After thermalcycling ageing, the elastic modulus of experimental cement decreased to 1.88 GPa, which was still markedly higher than that for the control group (1.46 GPa) (*p* < 0.05). The flexural strength of the experimental group was higher than that of the control group irrespective of 24 h or after ageing although there was no statistically significant difference. Shore hardness increased slightly after thermalcycling ageing in both two groups, but the difference was not statistically significant. In addition, the experimental group demonstrated higher Shore hardness (88.20 for the 24 h subgroup and 88.68 for thermalcycling ageing subgroup) than the control group (85.94 and 87.18, respectively).

**Table 1:** Results of mechanical tests of resin cement

Paste type	Control			Res@HMS modified cement		n
	24 h	Aged		24 h	Aged	
Elastic modulus (GPa)	1.59 (0.15) a	1.46 (0.21) a		2.24 (0.31) c	1.88 (0.24) b	10
Flexural strength (MPa)	110.67 (2.12) b	80.19 (1.49) a		115.73 (1.46) b	83.82 (1.14) a	10
Shore hardness	85.94 (1.94) a	87.18 (1.21) ab		88.20 (2.29) bc	88.68 (1.09) c	25

Data are shown as mean (standard deviation). Within the same row, means with same letter are not significantly different (*p* > 0.05)

### CCK8 and LDH assays of resin cement

**Fig. 7:**
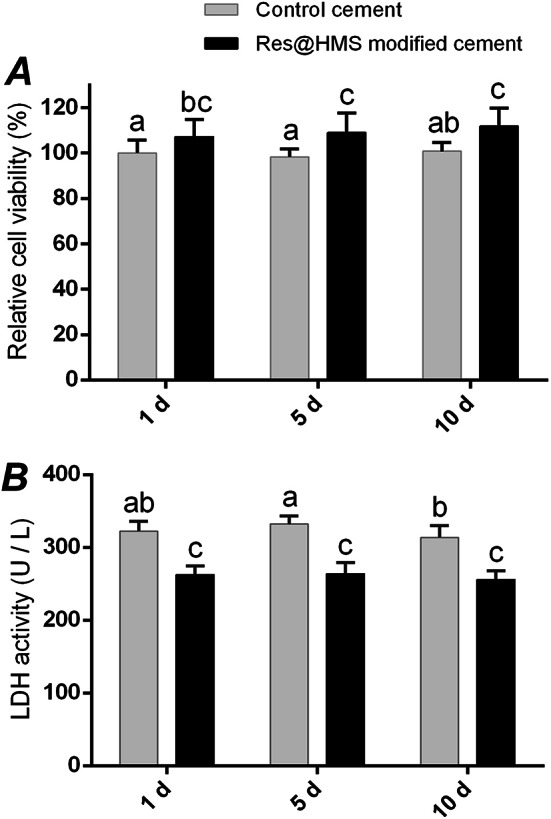
The relative cell viability (**A**) and LDH activity (**B**) of gingival fibroblast cultured by extracts from resin cements for 1, 5, and 10 days. Data are shown as mean ± standard deviation. Columns labeled with the same letters are not significantly different from each other (*P* > 0.05)

CCK8 and LDH assays were performed to evaluate the cytotoxicity of resin cement containing Res@HMS. The relative cell viabilities after culturing for 1, 5 and 10 d are displayed in Fig. 7A. Compared with control cultures, the extracts from Res@HMS-modified resin cement were less toxic after 24 h of exposure period because cell survival increased to 107%. The medium incubated with the experimental specimens for 5 and 10 d exhibited more favourable cytocompatibility than the other media because relative cell viability increased to 109% and 112%, respectively. The extracellular LDH, which widely exists in the cytoplasm and cell membranes and is immediately released into culture supernatants after cell damage, is another indicator of cell viability. The average LDH activity of the control group was 322.36, 332.14 and 313.88 U/L at 1, 5 and 10 d, respectively, whereas that of the experimental group was 262.44, 263.88 and 255.95 U/L respectively. LDH activity in the control group was more than 300 U/L on average, but it dropped to about 260 U/L at three different incubation time points in the experimental group (Fig. 7B).

### ROS measurement

**Fig. 8:**
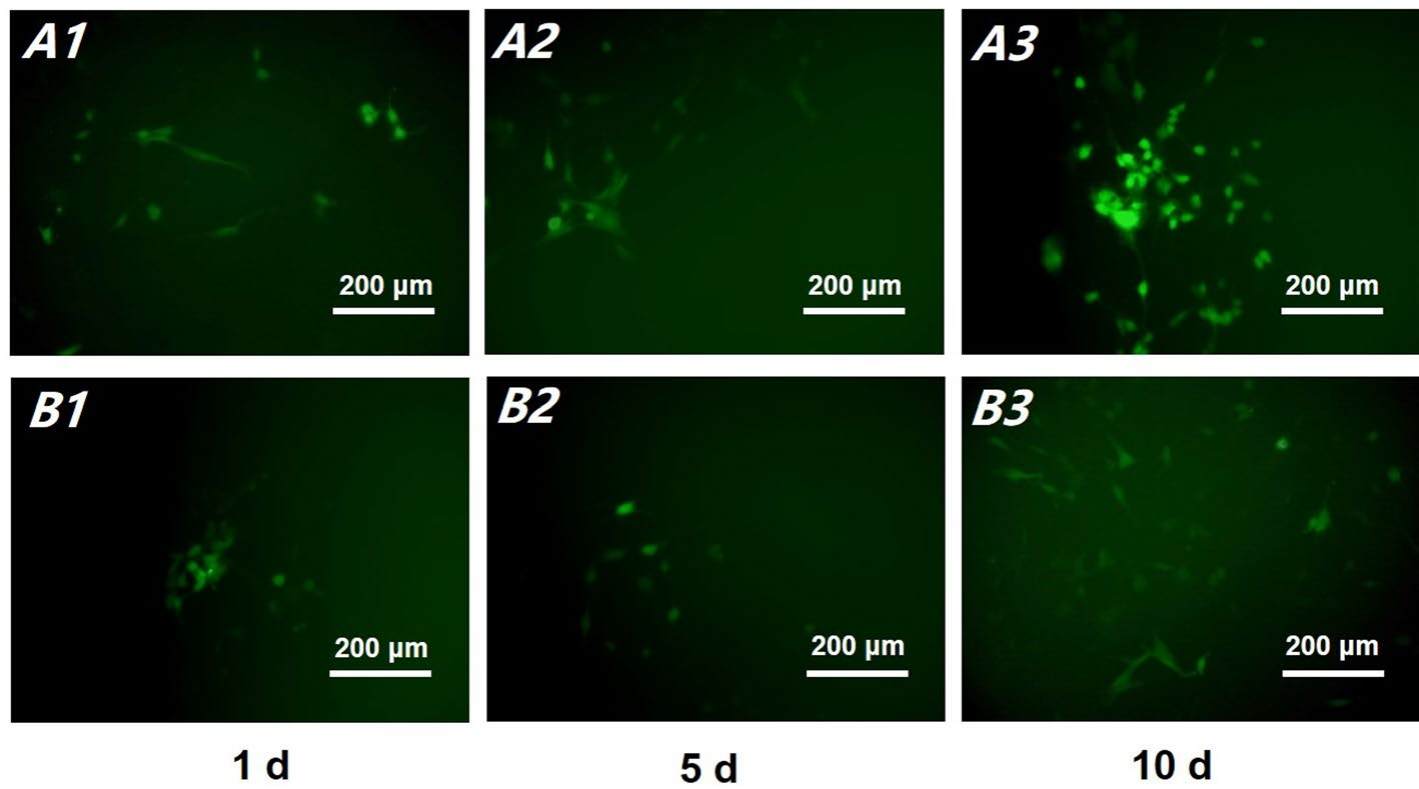
The representative fluorescence microscope images of intracellular ROS from the control (**A**) and Res@HMS modified cement extracts (**B**) groups. Cement extract medium were collected sequentially at time points of 1 (**A1**, **B1**), 5 (**A2**, **B2**) and 10 days (**A3**, **B3**), respectively

Given that resin monomers can induce glutathione depletion and ROS production [22], thus causing oxidative stress, cell dysfunctions, and apoptosis [23], ROS is assumed as an indicator of early cellular oxidative damage. Changes in ROS production are determined via DCF-DA assay in the present study. The presence of numerous ROS produces a bright DCF fluorescence. Resin cement-induced DCF fluorescence changes at 1, 5 and 10 d time points are plotted in Fig. 8. Compared with the control (Fig. 8A1–A3), cell incubation in Res@HMS-modified cement extracts evidently decreased ROS production (Fig. 8B1–B3). On 1 and 5 d, both groups produced a similar DCF fluorescence intensity, but the experimental group had fewer affected cells that emitted green fluorescence. After 10 d of incubation, the control group produced markedly higher fluorescence and had more affected HGFs than the experimental group (Fig. 8A3).

## Discussion

In the present study, we successfully established a novel strategy for bioactive modification of dental resin cement. Res@HMS was synthesized and subsequently incorporated into resin cement, resulting in a dental resin cement that exhibited satisfactory mechanical properties. Meanwhile, Res@HMS exerted efficient protective ability to human gingival fibroblasts against resin cement-induced cytotoxicity. Hence, both null hypotheses were rejected.

Despite significant advancements in materials and dentistry, there is still a long way to go in achieving a durable and stable resin cement. The greatest expectation for next-generation resin cement is to achieve ideal mechanical properties and reduce its toxicity. The introduction of mesoporous fillers has been proven to be an efficient way to improve the mechanical performance of polymer composites [7, 24]. In the present study, we synthesised HMS via an ammonia-catalysed sol–gel process. When polystyrene microspheres of uniform size were used as the templates, silica layers evenly coated and surrounded the polystyrene core, thereby forming particles with great dispersability. The porous structure of HMS was produced after removing the templates. The characteristics of HMS and Res@HMS evaluated via TEM, DLS, FTIR, TGA and BET–BJH demonstrated the effectiveness of the synthesis strategy proposed in this study. TEM revealed that HMS were typically spherical with diameter of about 1 μm (Fig. 3B) and a shell thickness of approximately 100 nm (Fig. 3D). Moreover, HMS had mesopores and a channel framework. The large hollow interiors of HMS were presumed to provide high drug loading space, and their mesoporous shells can realise sustained and controlled release of various drugs, such as resveratrol.

After the encapsulation process, the inner cavity and shell mesopores of Res@HMS became obscured and less clear than those of pure HMS (Fig. 3C). The average diameter of Res@HMS was 1186 nm (Fig. 4). FTIR spectroscopy and TGA curves (Figs. 5 and 6) revealed that the loading efficiency of resveratrol onto HMS was 12.49 wt%. This loading quantity percentage was higher than that of PO_3_- or NH_2_-functional mesoporous silica nanoparticles reported in other studies (always less than 10%) [25, 26]. After resveratrol loading, the average volume of Res@HMS became 0.08565 cm^3^/g, and its average size was 9.328 nm, implying that enough space was available for later infiltration of resin matrix.

A favourable mechanical performance of resin cement can considerably reduce the occurrence of restoration failure. The influence of Res@HMS on the mechanical properties of resin cement was clarified in the present study. Flexural strength, elastic modulus and surface hardness are basic features of dental restorative materials. The addition of Res@HMS remarkably enhanced the mechanical properties of experimental resin cement in 24 h (Table 1). There are likely two main factors accounting for the good mechanical performance. On one hand, Res@HMS, as rigid inorganic nanoparticles uniformly dispersed within the resin matrix, effectively transfers stress and restricts the movement of polymer chains. This conclusion aligns with recent report [27], which indicate that the elastic modulus of composite resins increases with higher filler concentration, regardless of the filler type. On the other hand, the mesoporous structure of Res@HMS enables resin monomers to infiltrate and form a mechanically interlocked interface, enhancing the elastic modulus through pore filling.

Intraoral temperature varies depending on dietary habits, and rapid temperature changes always challenge the stability of dental materials. Thus, the mechanical performance of Res@HMS-modified resin cement against thermocycling ageing must be evaluated. A previous study suggested that thermocycling between 5 °C and 55 °C for 10,000 cycles might represent 1-year service of restorative dental materials [28]. Accordingly, we adopted this model to assess the anti-ageing properties of the modified resin cement. After 10,000 runs of thermalcycling ageing, the elastic modulus of Res@HMS-modified cement decreased to 1.88 GPa, which was still markedly higher than that of the control group (1.46 GPa). This value of elastic modulus helps to decrease the prevalence of microleakages from the cement and therefore attenuate relative consequences, such as secondary caries and relocation of the restoration. Furthermore, Res@HMS substantially enhanced Shore hardness in both instant and ageing tests (Table 1). After the ageing test, Shore hardness slightly increased in both groups, which demonstrated consistent trend with previous investigations [29, 30]. Therefore, Res@HMS may strengthen the structure of resin matrix to protect against degradation, such as hydrolytic processes and temperature-related breakdown.

Since resin cement remain in contact with human cells for extended durations, minimizing their cytotoxicity is of utmost importance. As shown in Figs. 7 and 8, Res@HMS markedly attenuated the cytotoxicity induced by monomers leaching from the resin cement. Compared with that in the control group, the cell viability in the experimental group considerably increased at all time points. As the incubation time of extracts increased from 1, 5 d to 10 d, the cell survival rates of HGFs also increased (Fig. 7A). The increase in survival rate was primarily attributed to the protective effects of resveratrol as it was continuously released from the modified resin cement. Moreover, LDH is an oxidative enzyme that changes lactate into pyruvate during glycolysis [31]. LDH widely exists in the cytoplasm and cell membranes, and it is immediately released into culture supernatants following cellular damage. Therefore, the protective effects of Res@HMS on HGFs were evaluated by spectrometrically assessing the extracellular leakages of LDH. The LDH activity of the experimental group evidently decreased at three different incubation time points, indicating that the integrity of cell membrane was protected well and the addition of Res@HMS rescued resin cement-induced cellular damage (Fig. 7B).

Free monomers can induce glutathione depletion and ROS production, which would cause oxidative stress, cell dysfunctions, and DNA damage [23]. Compared with control group (Fig. 8A1–A3), ROS production evidently decreased in the HGFs after incubation with Res@HMS-modified cement extracts (Fig. 8B1–B3). Notably, ROS production increased with the incubation time of the control cement extracts. However, the Res@HMS-modified cement extracts induced substantially low DCF fluorescence irrespective of incubation time. Thus, monomer-induced cytotoxicity was effectively reduced by Res@HMS. This result was consistent with that of cell viability tests. Res@HMS could rescue monomer-induced cytotoxicity for a longer time. This result was also consisted with that of a previous study, which reported that resveratrol has a stronger and longer lasting ability over other antioxidants, such as quercetin and N-acetylcysteine, to enhance fibroblast proliferation and inhibit ROS production [32]. Resveratrol reportedly counteracts ROS production and lipid oxidation processes, either by suppressing the expression of AP-1 and NF-κB factors or by facilitating the generation of ferrous iron, carbon monoxide and biliverdin/bilirubin [33, 34]. These processes may contribute to the mechanism by which resveratrol exert cytoprotective effects. Therefore, Res@HMS-modified dental resin cements can help protect living cells from gingival tissues against ROS and oxidative damage.

Although this study has demonstrated the potential of Res@HMS as functional filler system for resin cement under laboratory conditions, its clinical translation still faces several critical uncertainties. Firstly, the long-term durability and functional sustainability of this filler system in the oral environment require further validation. Secondly, a comprehensive in vivo biosafety evaluation is urgently needed. Thirdly, quality control and cost management in scaled-up production may also pose significant challenges for industrialization. Finally, as an innovative composite system, the regulatory approval pathway for this material could encounter obstacles.

## Conclusion

In the present study, resveratrol-loaded hollow mesoporous silica (Res@HMS) was developed successfully. Results suggested that Res@HMS can serve as a novel delivery porous filler for dental resin cement. The effects of Res@HMS on the mechanical properties and cytotoxicity of resin cement were evaluated. This filler system can preserve the mechanical properties of resin cement against thermocycling ageing but also reduce resin monomer-induced cytotoxicity. This research demonstrated that the effective integration of mesoporous nanoparticles with plant extracts offers a novel filler that promises to balance the mechanical properties and cytotoxicity of resin cement, paving the way for developing a new generation of dental restorative materials.

## Data Availability

All data generated or analyzed during this study are included in this published article.
